# Pancreatic Cysts: Diagnostic Role of EUS-Guided Microforceps Biopsy and Confocal Laser Endomicroscopy

**DOI:** 10.1155/2019/3431048

**Published:** 2019-09-12

**Authors:** Darina Kohoutova, Sameer Zar, Rudolf Repak, Panagiotis Vlavianos, Jan Bures

**Affiliations:** ^1^The Royal Marsden Hospital NHS Foundation Trust, Fulham Road, Chelsea, SW3 6JJ London, UK; ^2^2nd Department of Internal Medicine-Gastroenterology, Charles University, Faculty of Medicine in Hradec Kralove, University Hospital, Sokolska 581, Hradec Kralove 500 05, Czech Republic; ^3^Hammersmith Hospital, Imperial College Healthcare NHS Trust, London, UK

## Abstract

Frequent use of high-quality cross-sectional imaging has led to a significant rise in diagnosis of pancreatic cystic lesions (PCLs). Despite the fact that enormous effort has been put into the research of PCLs within the last two decades and multiple guidelines have been developed, our clinical decision-making especially in regard to mucinous lesions remains limited. Currently, clinical assessment, cross-sectional imaging and EUS with fluid analysis (if appropriate) belong to the standard care in patients with PCLs. For differentiation of mucinous from nonmucinous cysts, the sensitivity of cytological investigation and CEA in the cyst fluid is 42% and 52-79%, respectively. Due to the limited accuracy, further diagnostic tools are warranted. Two EUS-guided approaches have been introduced recently. Through-the-(19-gauge EUS) needle Moray microforceps have been developed, and several studies have acknowledged their contribution to the correct diagnosis as they help to overcome limited cellularity of the EUS-guided cyst fluid aspiration and traditional cytology. Confocal laser endomicroscopy offers real-time images and seems to be a promising method for the diagnosis and differential diagnosis of pancreatic PCLs. Example images of the needle-based confocal laser endomicroscopy criteria for the diagnosis of PCLs have been suggested recently. Before both, Moray microforceps and confocal laser endomicroscopy can be widely accepted, further studies are necessary to determine the real diagnostic yield and the clinical efficacy.

## 1. Introduction

Frequent use of high-quality cross-sectional imaging has led to a significant rise in diagnosis of pancreatic cystic lesions (PCLs). The recent meta-analysis has confirmed pooled prevalence of 8% in asymptomatic individuals [1]. Incidence of PCLs increases with age and reaches 37% in patients aged >80 years [2]. It has been acknowledged that individuals with PCLs have a significantly higher overall risk of pancreatic cancer [3]; nevertheless, clinicians face a challenge how to optimize management of individuals with a PCL, when currently insufficient diagnostic tools are taken into account [4]. Patients should not be overtreated with surgery and on the contrary, individuals with a malignant PCL should not be kept under surveillance inappropriately [5].

The aim of our paper is to review classification of pancreatic cysts and to discuss the role of the most recent EUS- (endoscopic ultrasound-) guided diagnostic options for PCLs.

## 2. Classification of PCLs and Current Knowledge

Pancreatic cystic lesions are divided into mucinous lesions, including mucinous cystic neoplasm (MCN) and intraductal papillary mucinous neoplasm (IPMN) and nonmucinous lesions which include serous cystic neoplasm (SCN), pseudocyst, cystic neuroendocrine tumour, solid pseudopapillary tumour, and cystic pancreatic ductal adenocarcinoma [6–8]. Basic characteristics of PCLs are summarized in Table 1. Mucinous cystic lesions belong, together with pancreatic intraepithelial neoplasia, to the precursor lesions for pancreatic adenocarcinoma [9].

Patients with symptomatic PCLs can present with jaundice, recent onset of type 3 diabetes, (recurrent) pancreatitis, anorexia, weight loss, abdominal/back pain, nausea, and/or vomiting [7, 8]. Clinical assessment, cross-sectional imaging and EUS with fluid analysis, if appropriate (cytology, CEA), play the major role in current standard care in patients with a PCL. MRI/MRCP has been proven to be superior to CT in identifying communication between a PCL and the pancreatic ductal system and the presence of a mural nodule and in identifying if a patient has single or multiple PCLs [10–13]. In a recent meta-analysis, cytological investigation of the cyst fluid had 42% sensitivity and 99% specificity for differentiation of mucinous from the nonmucinous PCLs [14]. Cyst fluid CEA ≥ 192 ng/mL can differentiate mucinous from nonmucinous cyst with a sensitivity of 52-78% and specificity of 63-91%. Cytology and/or cyst fluid CEA level is not helpful in differentiation between MCN and IPMN [10, 15]. Despite the fact that the evidence is weak, antibiotic prophylaxis prior to an EUS-guided FNA of PCLs and 3-5 days after keeps being used routinely. Guarner-Argente et al. [16] and recently Facciorusso et al. [17] have not observed reduction of risk of infection after antibiotic prophylaxis. In view of this, further prospective studies are warranted with the aim to abandon routine periprocedural use of antibiotics.

### 2.1. Intraductal Papillary Mucinous Neoplasia (IPMN)

**Figure 1:**
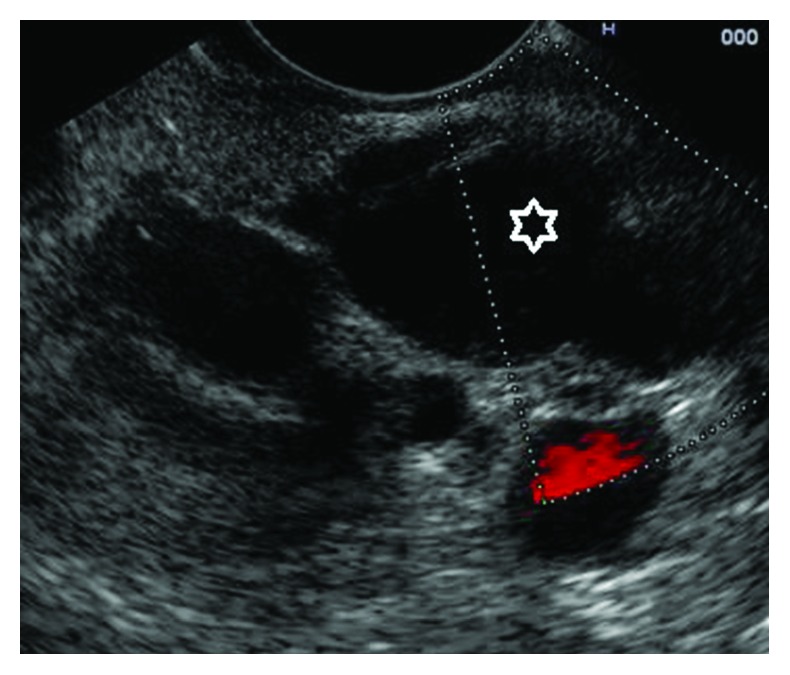
Main duct intraductal papillary mucinous neoplasia (asterisk: dilatation of the main pancreatic duct).

**Figure 2:**
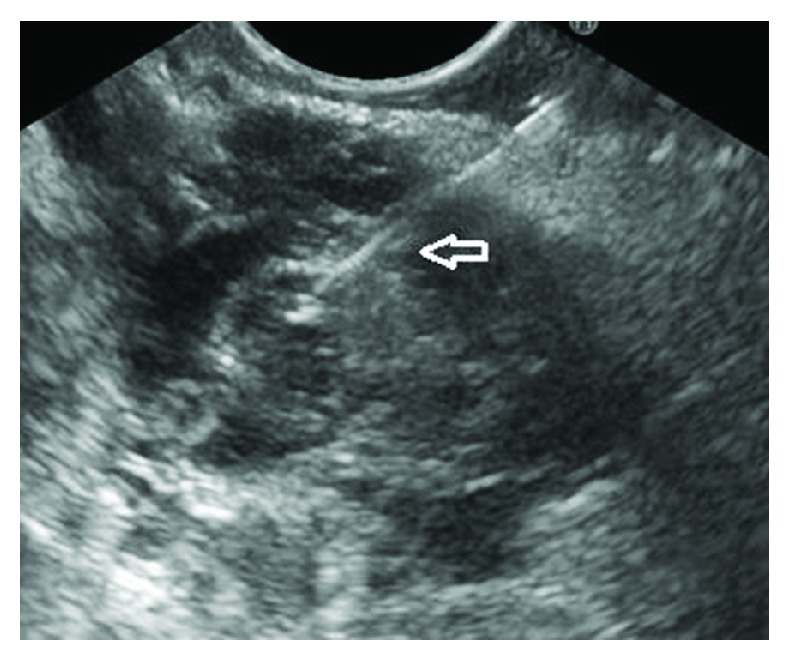
Main duct intraductal papillary mucinous neoplasia, FNA performed (arrow pointing at the FNA needle).

IPMNs are classified into the main duct (Figures 1 and 2), mixed type, and branch duct neoplasias according to the communication with and involvement of the main and/or branch pancreatic ductal system [9]. Majority of IPMNs are solitary and are localized in the pancreatic head, yet 20-40% are multifocal [18]. Typically, males (in 60-70%) of age 60-70 years would be diagnosed with an IPMN. The absence of capsule, communication with the pancreatic duct, presence of mucin, and cystic fluid high in CEA and amylase belong to the main features of IPMNs [19]. Based on histological characteristics and immunohistochemical reactivity for mucins (MUC), IPMNs are classified into gastric (less aggressive phenotype, usually originating in branch ducts), intestinal, pancreatobiliary, and oncocytic (more aggressive phenotypes, usually originating in the main pancreatic duct) types [9, 18, 20, 21]. Invasive carcinomas related to IPMN can be either colloid or tubular (conventional), and there is a clear evidence that the colloid carcinomas have a better prognosis than the tubular carcinomas [22–24]. The most typical mutations observed in IPMNs are those in oncogenes KRAS and GNAS and in tumour suppressor gene RNF43 [6]. Due to the risk of malignant transformation, patients with main duct IPMNs and mixed type IPMNs should be considered for surgery. According to the recent European guidelines, the absolute indications for surgery are tumour-related jaundice, solid mass, presence of an enhancing mural nodule (≥5 mm), positive cytology for malignant/high grade dysplasia, and/or main pancreatic duct dilatation ≥ 10 mm [10].

### 2.2. Mucinous Cystic Neoplasm (MCN)

**Figure 3:**
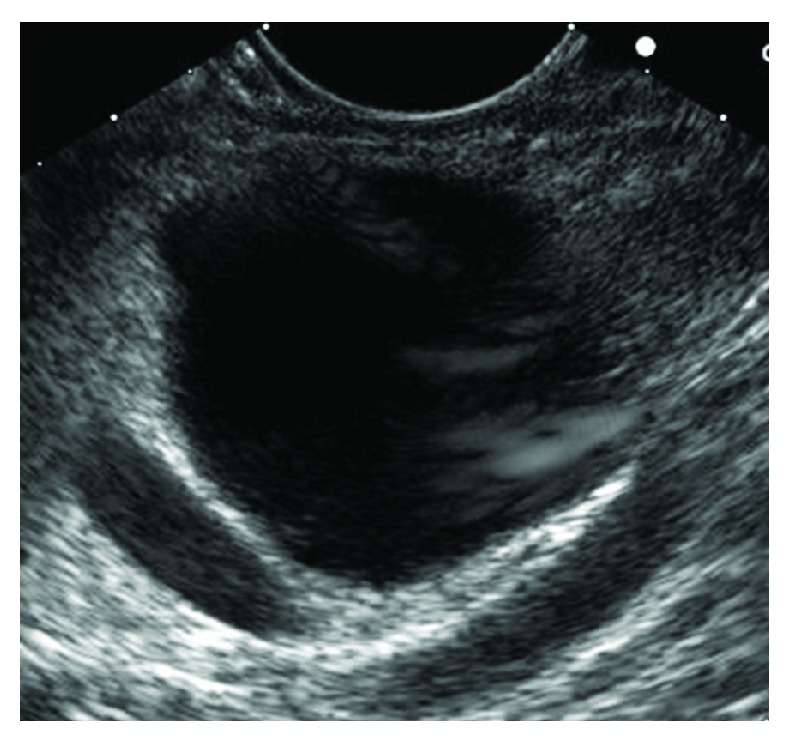
Mucinous cystic neoplasm. CEA: 242 ng/mL. Cytology benign.

MCNs are usually solitary large unilocular cysts predominantly found in the body or the tail of the pancreas in 40-50-year-old females (Figure 3). Cysts are characterized by the absence of communication with the pancreatic ductal system [19]. Peripheral “eggshell” calcification is seen in less than 20% of MCNs; nevertheless, such a finding is specific for a mucinous cystic neoplasm and is highly predictive of malignancy [25, 26]. Further typical features are the presence of ovarian-like stroma (with expression of hormone receptors) and mucin-producing epithelium. Cystic fluid is high in CEA and low in amylase [6, 25]. Mutation in KRAS oncogene is the most commonly found mutation; GNAS mutation is not observed in MCN—on contrary to an IPMN [27]. As MCN is usually discovered in younger patients in the body or the tail of the pancreas and has clearly a malignant potential, majority of centres will recommend surgery [25]. The recent European guidelines suggest resection for all patients with a MCN ≥ 40 mm in size or who are symptomatic or have risk factors (such as a mural nodule), irrespective of the size [10].

### 2.3. Serous Cystic Neoplasm (SCN)

**Figure 4:**
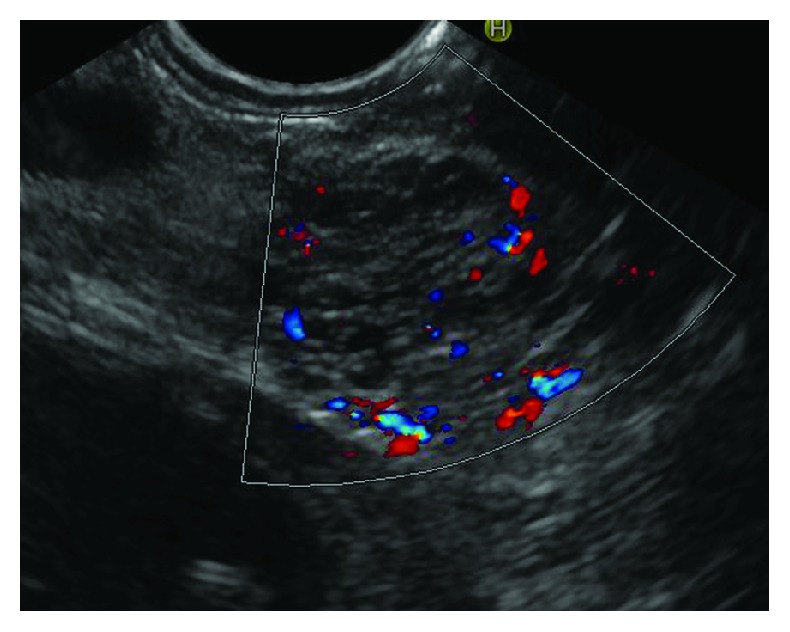
Serous cystic neoplasm with vascular septa.

**Figure 5:**
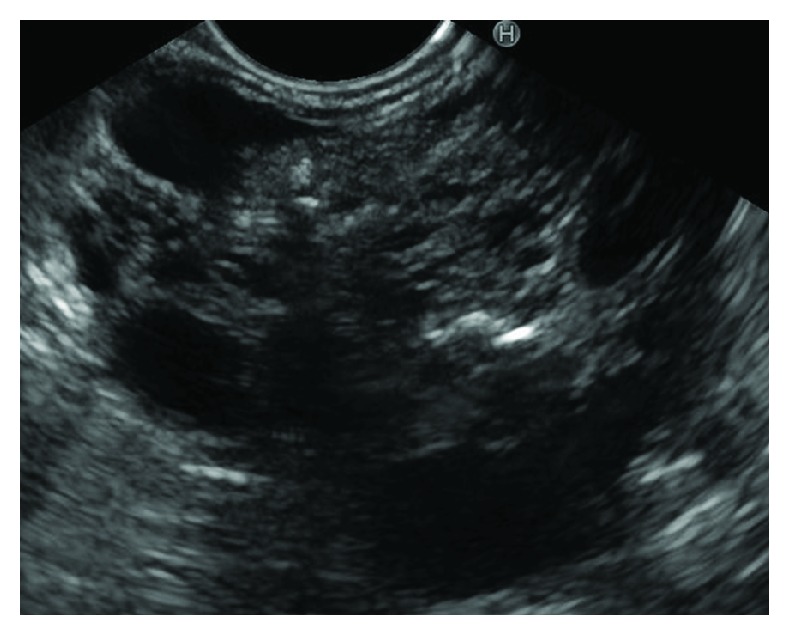
Serous cystic neoplasm with micro- and macrocysts.

SCN are benign cystic lesions predominantly found in the tail of the pancreas in females aged around 70 years (Figures 4 and 5). Imaging usually shows microcystic “honeycomb-like” lesion with central scar and central calcification with no communication with the pancreatic duct. The absence of mucin in the cyst, low CEA, and low amylase in the cyst fluid are characteristics of SCN [19, 25]. Mutations in VHL gene are typical for serous cystic lesions [28]. Surgical treatment should be proposed only if the diagnosis remains uncertain after a complete workup, if significant and related symptoms are present (jaundice, pancreatitis, and gastric outlet obstruction), or if exceptionally, a concern regarding malignancy arises [25, 29].

### 2.4. Pseudocyst

**Figure 6:**
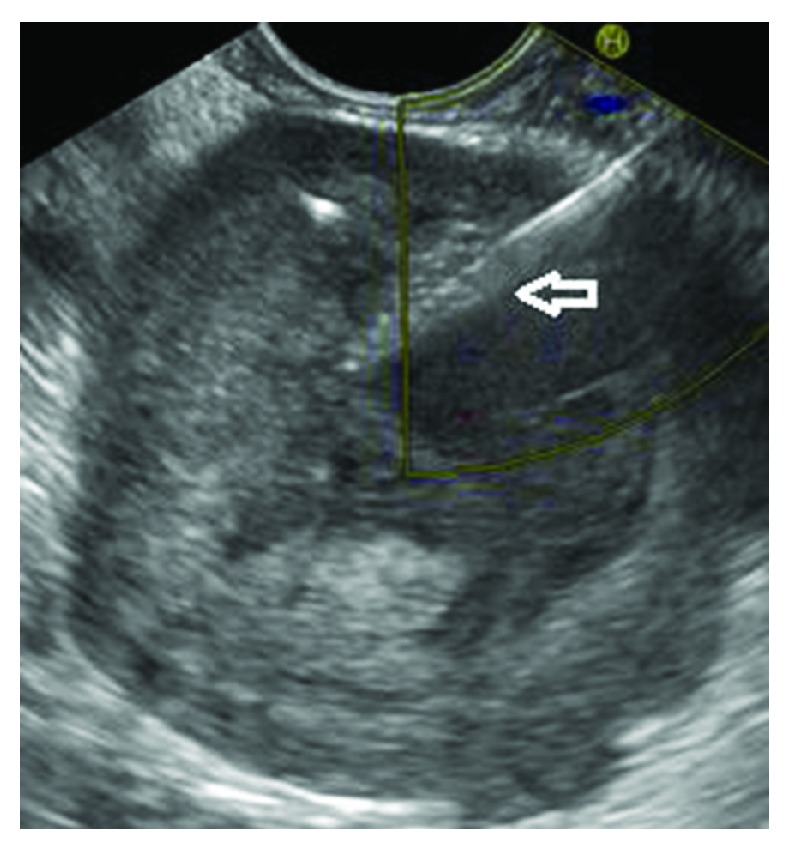
Pancreatic pseudocyst (FNA had been performed before the AXIOS stent was inserted; arrow pointing at the FNA needle).

**Figure 7:**
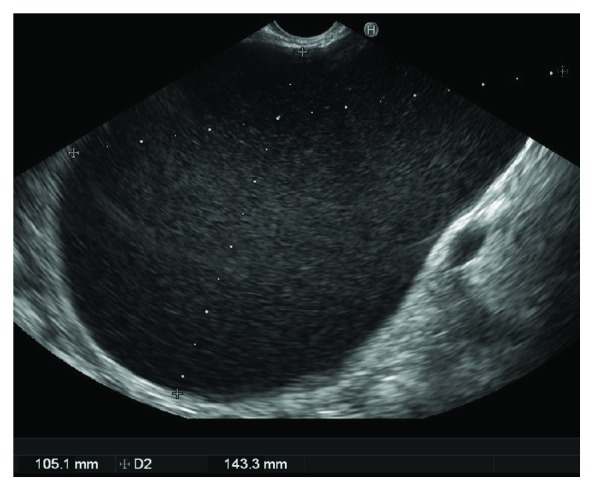
Large pancreatic pseudocyst (143 × 105 mm).

Pancreatic pseudocysts can be present anywhere in the pancreas and are more frequently found in males, usually in association with chronic pancreatitis (Figures 6 and 7). CEA is low, no mucin, and no molecular markers related to malignancy can be detected [6, 25]. Communication with the pancreatic duct is usual; therefore, the content is rich in amylase. Amylase <250 U/L (4.2 *μ*kat/L) may exclude the presence of a pseudocyst with a sensitivity 44% and specificity 98% [30].

### 2.5. Solid Pseudopapillary Tumour (SPT)

**Figure 8:**
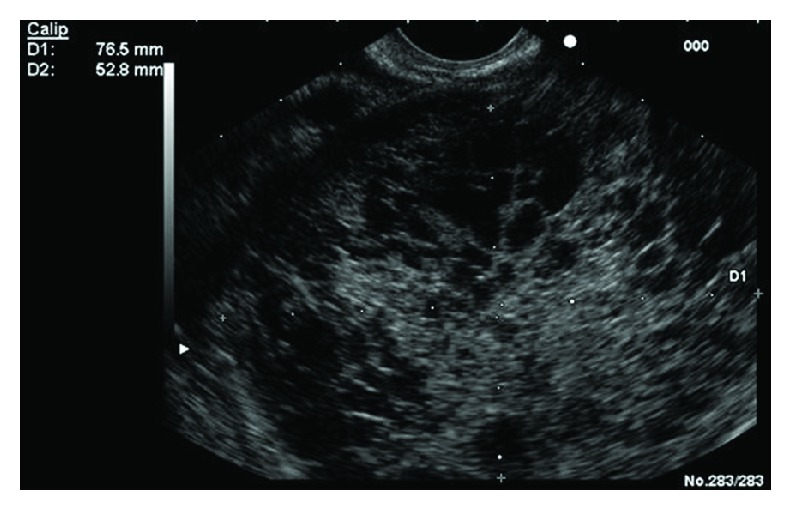
Solid-cystic pseudopapillary tumour.

SPTs, rare lesions, are typically identified in young females and express progesterone and estrogen receptors (Figure 8). They can be found anywhere in the pancreas and usually consist of mixed solid-cystic lesions. There is no communication with the pancreatic duct; the cyst fluid is low in CEA and amylase [6, 19, 25]. SPTs belong to slowly growing tumours with low malignant potential and infrequent metastases [31]. Surgical resection is warranted [25].

Enormous effort has been put into the research regarding pancreatic cystic lesions within the last two decades; nevertheless, diagnostic accuracy, as shown above, is rather poor. Multiple guidelines have been developed (including Sendai [32], Fukuoka [22], revised Fukuoka [33], American Gastroenterological Association (AGA) [34], and European guidelines [10]); still, our current clinical decision-making especially in regard to mucinous lesions remains limited. Further diagnostic tools are warranted. We offer an update on two EUS-guided methods, and we discuss their role in the PCL diagnosis.

## 3. EUS-Guided Microforceps Biopsy

In an attempt to improve the diagnostic yield of PCLs, through-the-needle direct intracystic biopsy and pancreatic cystoscopy were first performed in 2010 [35]. Biopsy forceps and a SpyGlass fiber optic probe were passed through the 19-gauge EUS needle in two patients with a PCL in the head of the pancreas. Diagnosis of a mucinous lesion was established in both cases. One patient developed severe acute pancreatitis one month after the biopsies, which was rather not associated with the procedure [35]. Another report came in 2015, when Barresi et al. documented a contribution of miniforceps biopsy to the diagnosis of a mucinous tumour in the body of the pancreas in a 46-year-old woman. Biopsies were taken from the wall of the cyst [36]. Subsequently, novel through-the-needle Moray microforceps have been developed, and Pham et al. reported a first successful biopsy of an intracystic nodule leading to a diagnosis of a mucinous cyst [37]. Further, authors have confirmed in individual cases that Moray microforceps can be useful in determination of the nature of the PCLs and can contribute to their management and risk stratification [38–41]. Moray microforceps are 230 cm in length with a jaw opening width of 4.3 mm and a sheath of 0.8 mm in diameter that easily passes through a 19-gauge EUS-FNA needle [38]. The role of Moray microforceps in the preoperative diagnosis has been acknowledged subsequently [42–44].

A first larger study, retrospective in design, which involved 27 patients with PCLs, was published in 2018: 14 patients with cysts located in the pancreatic head and/or uncinate process and 13 patients with cysts located in the body and/or tail of the pancreas were enrolled. Moray microforceps were passed through the 19-gauge needle under the EUS guidance, and 3-4 subsequent samples were taken from the cyst wall and placed into formalin. After completion of biopsies, cyst fluid was aspirated and sent for cytology and CEA level analysis. Microforceps biopsies were technically successful in all 27 cases and provided a pathology diagnosis in 24 of 27 cases. No periprocedural adverse event was recorded (including bleeding, infection, perforation, and pancreatitis). Overall, microforceps biopsy results changed the diagnosis in 7 patients; nevertheless, cytology provided a diagnosis of a mucinous cyst in 4/27, and these have not been detected by microforceps biopsies. The authors therefore concluded that Moray forceps could be a useful adjunctive tool, which would be complementary to existing EUS-FNA sampling protocols for PCLs [45]. Also in 2018, Basar et al. published data on 42 patients from a multicentre study: they confirmed that Moray microforceps biopsy was far superior to cytology in providing a specific cyst diagnosis [46]. A similar conclusion came from Zhang et al.: pancreatic cyst fluid analysis and microforceps biopsy have comparable results in distinguishing between mucinous and nonmucinous cysts and for detecting high-risk cysts; nevertheless, similar to the study performed by Basar et al. [46], microforceps biopsy has been superior for diagnosing specific cyst subtypes [47]. Another most recent study published on microforceps biopsy by Kovacevich et al. has shown promising results, nevertheless, three adverse events (11%) have been recorded [48].

In conclusion, dedicated through-the-needle Moray microforceps allow biopsy of the PCL wall or a mural nodule. This helps to overcome the limited cellularity of the EUS-guided cyst fluid aspiration and traditional cytology. Further, it can provide guidance when at best modest accuracy of CEA is taken into account. Yet, the precise role of microforceps biopsy remains to be defined by large prospective studies before routine clinical implementation is recommended.

## 4. Confocal Microscopy

The principle of confocal laser scanning microscopy is not new; it was invented as early as 1957 [49]. Subsequent use in gastroenterology started in the mid-1990s [50]. The first generation of dedicated endoscopes enabled the introduction of confocal laser endomicroscopy (CLE) in the late nineties. A confocal endomicroscope was miniaturised to a size that made it possible to be integrated in the distal end of a high-resolution videoendoscope. A lot of research work was done afterwards, both experimental and clinical [51]. CLE classification was suggested for Barrett's oesophagus and colorectal neoplasia [51, 52]. Our group studied experimental pharmacokinetics, and organ distribution of fluorescein determined the optimum time interval for diagnostic scanning (5-10 minutes after the fluorescein administration) and found high concentration in all organs of the gastrointestinal tract (including the pancreas), necessary for optimal confocal laser imaging [53]. Recently, single miniaturised CLE probes have become commercially available. These probes can be introduced through a working channel of a conventional videoendoscope into the lumen of the gastrointestinal tract or through a 19-gauge needle for fine-needle-based CLE. They enable observation of the inner wall of pancreatic cystic lesions during an endoscopic ultrasound-guided fine-needle aspiration [6, 51, 54–58].

Since 2010, more than fifty papers have been published, including ESGE [54, 59] and ASGE technology reviews [51] and a meta-analysis [60]; however, only few clinical studies have been accomplished so far [61–68]. Konda et al. [61] published their first experience with a prototype confocal laser probe. Eighteen patients (with 16 cysts and 2 mass lesions) were investigated in this multicentre feasibility study at a tertiary setting. CLE was technically feasible (in 17 of 18 cases) using a 19-gauge needle under EUS guidance. There were no device malfunctions; two cases were complicated with acute pancreatitis. The diagnosis was confirmed with histology or positive cytology in 10 out of 18 patients [61]. The INSPECT study investigated 66 patients (images were available for 65) in USA, Germany, and France. The authors aimed to define criteria for differentiation of mucinous and nonmucinous cystic lesions. An epithelial villous structure on confocal images was associated with mucinous cysts significantly [62]. Eighteen patients with indeterminate pancreatic duct strictures were investigated prior to surgery in another study [63]. Real-time CLE images were obtained during ERCP. Cytology or histopathology in 15 of 16 cases showed similar results to CLE interpretation. Agreement between cytology or histopathology and CLE was high (*κ* = 0.8). Pancreatic CLE changed management in four patients [63]. The DETECT study combined EUS-guided through-the-needle direct visualisation (SpyGlass fiber optic probe) and probe-based CLE inserted through a 19-gauge needle. Thirty patients with pancreatic cystic neoplasms were enrolled. The combination of cystoscopy and CLE of pancreatic cysts might have strong concordance with the clinical diagnosis of pancreatic cystic neoplasms (sensitivity 87, specificity 77, and positive and negative predictive values 100%) [64]. Napoleon et al. [65] investigated 31 patients with pancreatic cysts and identified criteria for the diagnosis of serous cystadenoma [65]. Twenty patients with pancreatic cystic neoplasms were investigated within a 16-month period in another study [66]. The procedure and confocal image acquisition were successful in 90%. The sensitivity, specificity, and diagnostic accuracy were 66, 100, and 80%. No complications were recorded [66]. The pancreatic cyst epithelial wall can be visualised successfully by CLE also in ex vivo surgical specimens [67]. Krishna et al. [68] investigated ten patients for the reproducibility of the in vivo endoscopic ultrasound-guided needle-based CLE image patterns in an ex vivo setting. Both in vivo (preoperative) and ex vivo confocal laser imaging of the surgically resected pancreatic cystic lesions correlated with surgical histopathology [68].

**Figure 9:**
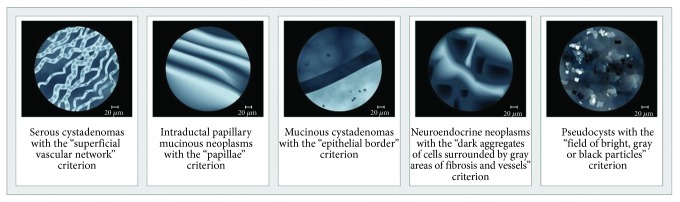
Confocal laser endomicroscopy for evaluation of pancreatic cystic lesions. Adopted from Napoleon et al. [65], Napoleon et al. [56], and Kadayifci et al. [66]. Drawing: Hana Kotlandova.

Example images of the needle-based CLE criteria for the diagnosis of PCLs have been suggested: (a) serous cystadenomas with the “superficial vascular network” criterion; (b) intraductal papillary mucinous neoplasms with the “papillae” criterion; (c) mucinous cystadenomas with the “epithelial border” criterion; (d) neuroendocrine neoplasms with the “dark aggregates of cells surrounded by gray areas of fibrosis and vessels” criterion; and (e) pseudocysts with the “field of bright, gray, or black particles” criterion [56, 65, 66] (Figure 9). For the near future, it will be mandatory to set a validated interpretation system of the CLE of pancreatic cystic lesions, to establish a unified training programme and to create a close standardized cooperation of gastroenterologists/endoscopists and pathologists. There is conflicting data on the reproducibility and accuracy of needle-based CLE in the available literature. In a multicentre US study, interobserver agreement of needle-based CLE recordings of 15 patients was low; the mean accuracy of observers was only 46%. Interobserver agreement for the final diagnosis was poor (*κ* = 0.13) [69]. According to another project [70], interobserver agreement, intraobserver reliability, and diagnostic accuracy were high in differentiation of mucinous versus nonmucinous pancreatic cystic lesions. In a study with 29 consecutive patients (between 2013 and 2016), the overall sensitivity, specificity, and accuracy for the diagnosis of mucinous lesions were 95%, 94%, and 95%, respectively. The interobserver agreement and intraobserver reliability were high (*κ* = 0.81 and *κ* = 0.86, respectively). Similar results were achieved for recognizing the characteristic image pattern of serous cystadenomas (*κ* = 0.83 and *κ* = 0.85). The overall specificity, sensitivity, and accuracy for the diagnosis of serous cystadenomas were 99%, 98%, and 98%, respectively [70].

Safety of needle-based CLE refers either to EUS-guided puncture of the cyst lesions or to side effects of i.v. fluorescein administration. Acute pancreatitis can complicate up to 4% of procedures; bleeding associated with a needle puncture is another possible complication [65]. A series of 2272 patients with CLE revealed no serious adverse complications of i.v. fluorescein administration. Mild adverse events (in 1.4%) included nausea, vomiting, transient hypotension, diffuse rash, and mild epigastric pain [71].

Financial aspects must be also considered. Needle-based CLE of pancreatic cyst lesions increases the cost of the diagnostic process significantly [51]. On the other hand, needle-based CLE used with EUS-based fine-needle aspiration and/or biopsy might improve diagnostic accuracy, helping to reduce unnecessary surgery and patient follow-up, thus resulting in significant economic benefits by reducing the incidence of misdiagnosis owing to improved diagnostic accuracy [72]. Before the technology can be widely accepted, further studies are necessary to determine the real clinical efficacy and to evaluate the cost-effectiveness [51].

CLE seems to be a promising method for the diagnosis and differential diagnosis of pancreatic cystic lesions. Nevertheless, the common use is limited by its high cost and need of specific operator training (as a standardized institutional programme). That is why CLE is not easily available yet. Further studies are needed to evaluate the real diagnostic yield, cost-effectiveness, and health care economic analyses prior to implementation into a routine clinical practice.

## 5. Conclusions

Based on the current clinical practice, diagnostic accuracy for pancreatic cystic lesions is modest at best. Further diagnostic tools are warranted. Two EUS-guided methods have been introduced recently: Moray microforceps and CLE. They seem to be promising in regard to diagnostic yield; nevertheless, further studies are warranted to determine clinical efficacy and to evaluate the cost-effectiveness.

## Figures and Tables

**Table 1:** IPMN: intraductal papillary mucinous neoplasm; MCN: mucinous cystic neoplasm; SCN: serous cystic neoplasm; SPN: solid pseudopapillary neoplasm; p: predominantly; HOP: head of pancreas.

	IPMN	MCN	SCN	Pancreatic pseudocyst	SPN
Male	60-70%	5%	10%	75%	10%
Age (years)	60-70	40-50	70	Around 50	30
Localization	p HOP	p body and tail	p tail	Any localisation	Any localisation
Communication with PD	Yes	No	No	Yes	No
Cytology	Mucinous cells	Mucinous cells		Inflammatory cells	
CEA in cyst	High	High	Low	Low	Low
Mucin in cyst	Yes	Yes	No	No	No
Amylase in cyst	High	Low	Low	High	Low
